# A Low Redundancy Wavelet Entropy Edge Detection Algorithm

**DOI:** 10.3390/jimaging7090188

**Published:** 2021-09-17

**Authors:** Yiting Tao, Thomas Scully, Asanka G. Perera, Andrew Lambert, Javaan Chahl

**Affiliations:** 1 UniSA STEM, Mawson Lakes Campus, University of South Australia, Adelaide, SA 5095, Australia; asanka.perera@mymail.unisa.edu.au (A.G.P.); Javaan.Chahl@unisa.edu.au (J.C.); 2 School of Engineering and Information Technology, University of New South Wales, Canberra, ACT 2610, Australia; tscully@fortinet.com (T.S.); a.lambert@adfa.edu.au (A.L.); 3 Joint and Operations Analysis Division, Defence Science and Technology Group, Melbourne, VIC 3207, Australia

**Keywords:** edge detection, wavelet decomposition, Shannon entropy

## Abstract

Fast edge detection of images can be useful for many real-world applications. Edge detection is not an end application but often the first step of a computer vision application. Therefore, fast and simple edge detection techniques are important for efficient image processing. In this work, we propose a new edge detection algorithm using a combination of the wavelet transform, Shannon entropy and thresholding. The new algorithm is based on the concept that each Wavelet decomposition level has an assumed level of structure that enables the use of Shannon entropy as a measure of global image structure. The proposed algorithm is developed mathematically and compared to five popular edge detection algorithms. The results show that our solution is low redundancy, noise resilient, and well suited to real-time image processing applications.

## 1. Introduction

Edges of an image are considered to be a low-level image feature. They are undoubtedly one of the most important features in an image. Edge detection research spans several decades but still remains as an active research area due to its applicability to a wide range of computer vision tasks.

In the literature, a range of edge detection techniques have been developed and used by the computer vision community. Edge detection techniques are extensively used in many application areas such as outlier detection in medical images [1], remote sensing [2], image-to-image translation [3], photo-sketching [4], optical character recognition [5], and robotic vision [6].

Edge detection is a fundamental step in image processing. Often, edge detection is the first step applied to an image field in preparation for further image processing operations. Edge detection allows an image field to be segmented to extract important features, reducing redundancy in an image field and enabling subsequent image processing task complexity to be significantly reduced. Robust and computationally efficient techniques are therefore central to many image processing applications.

An edge is defined as the change of intensity within a given image field I(x,y). Edges within I(x,y) are described using gradient magnitude. Whilst this definition of an edge is simplistic, identification and extraction of edges are a non-trivial task. The non-trivial nature of edge detection and the importance to subsequent image processing tasks has resulted in edge detection being an active area of research for decades [7,8,9,10,11]

Multiple edge detectors have been developed with no single edge detector algorithm providing the ultimate solution across all image scenarios. Prevalent edge detection methods such as Canny [12], Prewitt [13], Roberts [14], Sobel [15] and Zerocross [16] have proven popular due to their efficiency and ease of implementation, making them useful for real world image processing applications [11,17,18].

In this paper, we propose a new edge detection method based on the coupling of wavelet and entropy based techniques. This new edge detection algorithm capitalizes on the algorithmic efficiency of wavelet based techniques while incorporating entropy techniques to select the most appropriate wavelet scale to analyze for edges. Wavelet decomposition is used to extract edge location and orientation data across multiple frequency domains. Entropy is then used to assess the multi-scale decomposition data and determine which wavelet decomposition level contains the greatest image structure. This process produces a low redundancy edge detection output image with a significant increase in computational efficiency. In doing so, the new method is well suited to implementation in real-time image processing applications.

This paper is organized as follows: closely related work has been discussed in Section 2. Section 3 describes the proposed algorithm in detail. Section 4 provides experimental results and analysis of our solution when compared to five widely used edge detection algorithms. The computational efficiency and the resilience of the approach to noise was analyzed. Section 5 concludes the study.

## 2. Related Work

Several works have been published in the edge detection area. A complete list of studies can be found in these surveys [7,19]. For ease of analysis, in the following sub-sections, we broadly categorize edge detection algorithms based on their design.

### 2.1. First Order

Prewitt, Roberts and Sobel edge detection techniques are first order differential image based filters. Therefore, each filter has strengths and weaknesses when detecting edges in a scene. Thus, no single first order filter provides a generalized edge detection solution for all image scenarios [20,21]. First order methods typically perform poorly when the image contains a high noise content [22]. Furthermore, these operators are typically computationally expensive as they require convolution to enable implementation.

### 2.2. Second Order

A number of second order differential methods [16,22,23,24,25] exist for the detection of edges. Second order differential methods detect the crossing of the zero gradient axis of $\nabla^{2}I\left(x,y\right)$ to define the location of edges within an image. As a result, second order differential methods are sensitive to noise within the image, making them less robust [22]. To increase robustness, second order differential methods typically employ filtering, which in turn results in loss of edge data contained within the image.

The Canny Edge Detector is accepted as the industry standard for edge detection techniques [22]. John Canny [12] aimed to create an optimized edge detection technique that satisfied a comprehensive set of goals for the computation of optimal edge points. The Canny Edge Detector succeeded in creating a robust and computationally efficient edge detection technique; however, it relies heavily on image smoothing to reduce noise within images. Smoothing is typically achieved through Gaussian filtering of the image, resulting in degradation of edge data. As a result, the Canny Edge Detector can break down when the gradient magnitude is small in comparison to the noise present within the image [22]. An approximation of Canny edge detection has been developed by Deriche [26] and by Van Vleit et al. [27]. Deriche proposed an extension to the two-dimensional (2D) case (Canny’s optimal edge detector), with the resulting filtering structures implemented as 2D infinite impulse response (IIR) digital filters.

### 2.3. Entropy Based

Entropy based methods have also been investigated as a more robust and computationally efficient method for edge detection [28,29,30,31,32,33,34,35,36]. Entropy based methods apply the concept of information entropy [37] to determine whether structured edges are present. Entropy based methods typically calculate edges through thresholding based on evaluation of a global entropy value [28,29,38] or through comparison of localized entropy calculations with I(x,y) [30,32,34,35]. Entropy based methods assume that the calculated entropy value will be representative of the amount of structure present within the image. Entropy based methods have proved to be more resilient to noise within an image field while being computationally efficient compared to gradient based methods such as Prewitt, Roberts, Sobel, and Zerocross [22,32,36].

### 2.4. Wavelet Based

Wavelet based methods have been investigated as an efficient edge detection algorithm [39,40,41,42,43,44,45,46,47,48,49,50]. First and second derivative methods are based on Finite Impulse Response (FIR) and Infinite Impulse Response (IIR) filter design which can be computationally expensive to implement digitally. Wavelet based methods offer a method known as sub-band coding to enable shorter filter designs paired with subsampling to achieve more efficient computation than traditional Fourier based methods [51]. Multiscale analysis of wavelet decomposition levels enable more advanced techniques to be applied in the analysis of edge components within a given image.

### 2.5. Deep Learning/Machine Learning Based

In the past few years, deep learning based edge detectors have been proposed and developed. Because some Deep Learning (DL) methods are capable of predicting edges with more efficiency and accuracy, it has become popular in this field. More recently, DL methods that use convolutional neural networks (CNNs) to achieve the boundary and contour detection tasks have become popular, including some well known CNN based methods like HED [10], RCF [17], DeepEdge [52], DeepContour [53], and BDCN [54]. For instance, the HED approach offers image-to-image training and prediction, which produces the predicted edge map image directly [10]. Richer Convolutional Features (RCF) train a network using multi-scale and multi-level information to achieve contour prediction [17]. While deep learning-based methods remain state-of-the-art in the edge detection area, they need an extensive amount of work and resources for training and running compared to classical edge detection algorithms.

## 3. Methodology

**Figure 1:**
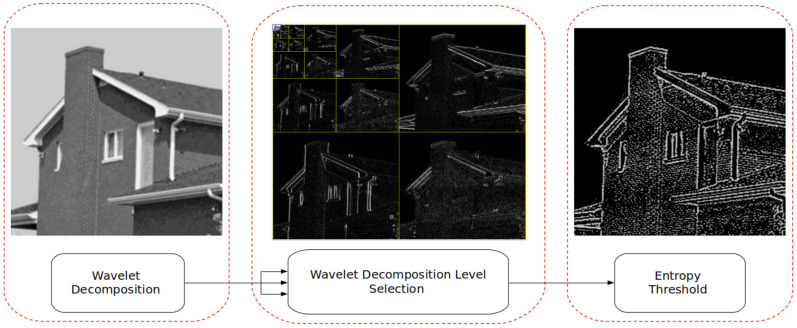
The overview of the proposed method.

In this section, we present the development steps of the method. We aimed at developing our algorithm to be efficient, low redundancy, and noise resilient. The proposed algorithm (LRWEEDA) consists of three distinct steps as shown in Figure 1.

For an image I(x,y), the edges within the image can be described using gradient magnitude $\left|\nabla I\left(x,y\right)\right|$ and gradient direction γ:

(1) $$\left|\nabla I\left(x,y\right)\right|=\sqrt{{\left(\frac{\partial I}{\partial x}\right)}^{2}+{\left(\frac{\partial I}{\partial y}\right)}^{2}}$$

(2) $$\gamma =∠\left(\frac{\partial I}{\partial x},\frac{\partial I}{\partial y}\right)$$

With regard to Equations (1) and (2), an edge is a collection of points with a similar gradient magnitude and direction clustered within a similar region. This implied definition means that edges within a given I(x,y) can be identified using three measures: image frequency to identify gradient magnitude, feature orientation to identify gradient direction, and image structure to identify clustering. We use wavelet filtering in combination with Shannon entropy to enable the identification of image frequency, feature orientation and image structure. The combination of wavelet filtering with Shannon entropy provides a simple, efficient and low redundancy method for edge detection.

Generally, entropy measures are considered unsuitable for detecting image structure such as edges [28,55]. This is due to entropy being based on the statistical properties and does not take into account the spatial properties of pixels within the image. Attempts have been made by [28,29,30,31,32] to overcome this by using multiple techniques including calculating localized entropy with windowed image sections and the use of relative entropy measures [35]. As a result, these methods are typically computationally intensive and their performance is sensitive to the window size used to calculate the localized entropy. Wavelet filtering enables an image field to be decomposed into individual frequency and orientation layers. In doing so, the output wavelet decomposition makes an inherent assumption of structure. Shannon entropy can be applied to each of these wavelet decomposition levels to give a quantitative assessment of image structure, or in our case, a quantitative assessment of edge structure. The following sections will explore in detail each of the steps shown in Figure 1.

### 3.1. Wavelet Decomposition

**Figure 2:**
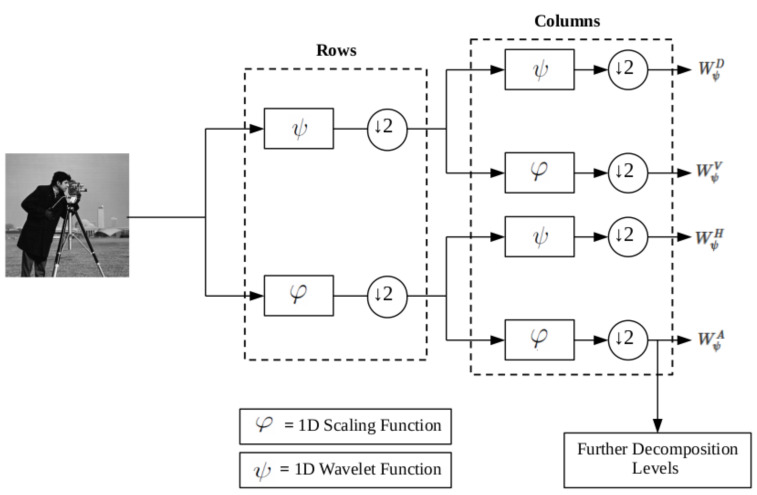
Application of wavelet decomposition to I(x,y).

Wavelets have received widespread interest over the past few decades with their ability to perform spatio-temporal analysis on signals of interest [55,56]. Their mathematical properties have enabled efficient implementations for high bandwidth applications using sub-band coding techniques. The one-dimensional, (1D) Continuous Wavelet Transform (CWT) is defined as

(3) $$W_{\psi \left(s,\tau \right)}=\int_{-\infty }^{\infty }f\left(x\right)\psi_{s,\tau }\left(x\right)dx,$$

where

(4) $$\psi_{s,\tau }\left(x\right)=\frac{1}{\sqrt{s}}\psi \left(\frac{x-\tau }{s}\right),$$

and *s* and τ are called scale and translation parameters. The scale parameter enables isolation of frequency components within a signal and τ enables the isolation of scale (frequency) components within time. For image processing applications, I(x,y) is defined as a discrete two-dimensional (2D) signal. To do so, Equations (3) and (4) must be represented in their discrete form and applied to I(x,y) as shown in Figure 2.

Figure 2 identifies that the 2D Discrete Wavelet Transform (DWT) is constructed through the application of cascaded 1D DWT filter banks applied to the rows and columns of I(x,y), respectively. The product of the cascaded 1D DWT filter banks produces directionally sensitive wavelets that measure image gradient magnitude tuned to a particular size. Figure 2 can be described using the following equations. Here, *i = H,V,D* and *j* is the decomposition level:

(5) $$\psi^{A}\left(x,y\right)=\varphi \left(x\right)\varphi \left(y\right)$$

(6) $$\psi^{H}\left(x,y\right)=\psi \left(x\right)\varphi \left(y\right)$$

(7) $$\psi^{V}\left(x,y\right)=\varphi \left(x\right)\psi \left(y\right)$$

(8) $$\psi^{D}\left(x,y\right)=\psi \left(x\right)\psi \left(y\right)$$

(9) $$\varphi_{j,m,n}\left(x,y\right)=2^{j/2}\varphi \left(2^{j}x-m,2^{j}y-n\right)$$

(10) $$\psi_{j,m,n}^{i}\left(x,y\right)=2^{j/2}\psi^{i}\left(2^{j}x-m,2^{j}y-n\right),\;i=H,V,D$$

(11) $$W_{\varphi (j_{o},m,n)}^{A}=\frac{1}{\sqrt{MN}}\sum_{x=0}^{M-1}\sum_{y=0}^{N-1}f\left(x,y\right)\varphi_{j_{0},m,n}\left(x,y\right)$$

(12) $$\begin{array}{c} W_{\psi (j,m,n)}^{i}=\frac{1}{\sqrt{MN}}\sum_{x=0}^{M-1}\sum_{y=0}^{N-1}f\left(x,y\right)\psi_{j,m,n}^{i}\left(x,y\right) \end{array}$$

The wavelet decomposition process is well suited to real-time applications as it can be implemented in an efficient parallel way with the use of short cascaded FIR filter banks, making it computationally efficient [57].

**Figure 3:**
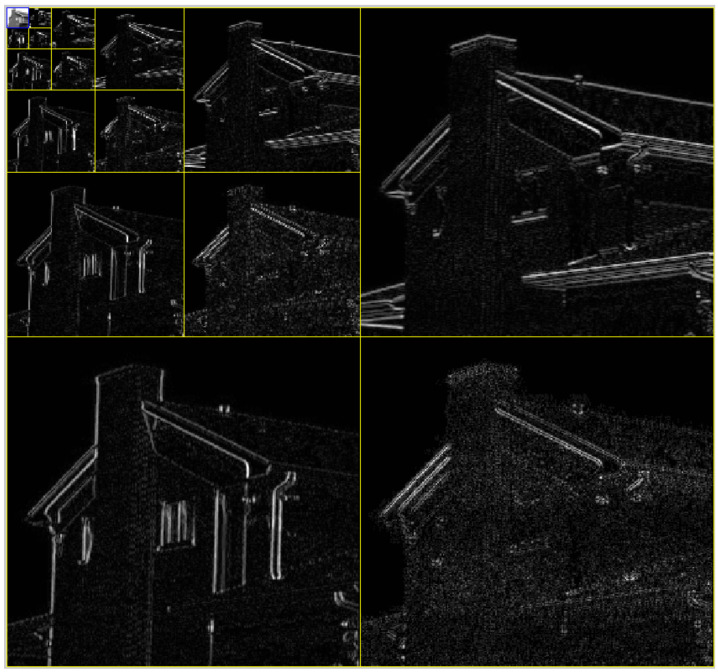
Wavelet decomposition applied to a test image. Six decomposition levels were applied.

The algorithm takes a grey scale image as the input (8 bit grey scale image with 512 × 512 pixels in size) and passes it through a 2D wavelet decomposition (six decomposition levels) using the Coiflet and Haar wavelet functions. In doing so, the image is broken down into individual frequency and orientation layers, isolating important image features according to their frequency and orientation within the image as shown in Figure 3.

Figure 3 shows that the wavelet decomposition has identified edge features of differing gradient magnitudes aligned in three different orientations: vertically, horizontally and diagonally. For each wavelet decomposition level, vertical and horizontal components are combined and normalized between zero and one to define ℧(j,m,n),

(13) $$℧\left(j,m,n\right)=normalise(W_{\psi }^{H}\left(j,m,n\right)+W_{\psi }^{V}\left(j,m,n\right))$$

where *j* represents the decomposition level. Each decomposition level can be interpreted as an image frequency band, where lower levels represent higher frequencies and higher levels represent low frequencies. Diagonal features within each level are discarded as they typically contain noisy information that will reduce edge feature quality [55].

Upon calculation of Equation (13), the algorithm successfully isolates all gradient magnitudes and directions into individual image frequency and orientation layers. This means that each decomposition level shown in Figure 3 has an assumed level of image structure. Dependent on the image input, different decomposition levels will indicate greater structure than others. Decomposition levels with greater levels of structure will represent greater value in the identification of edges. Decomposition levels with less structure offer less value in the identification of edges and can be discarded to optimize the edge detection algorithm.

Ultimately, to identify all edges within an image, all decomposition levels would be combined. However, when viewed from an optimization and redundancy viewpoint, we may be able to ignore or not include some edge features contained within specific decomposition levels resulting in only minor degradation to the final edge detection solution. This is analogous to lossy image compression schemes or message redundancy problems [55,58]. The algorithm assumes that the decomposition level with the most structure can be assumed to have the most useful edges within it and therefore should be used for further image processing applications. To quickly measure the amount of structure in each decomposition level, we use Shannon entropy for structure measurement.

### 3.2. Wavelet Decomposition Level Selection

After an image has been broken down into individual scale (frequency) and orientation layers using the wavelet decomposition step, the most suitable level must be selected for further processing. Shannon entropy is used to determine the most suitable decomposition level for the detection of edges. Shannon entropy is typically unsuitable as a measure of image structure [55] as it only accounts for the statistical distribution of pixel values, as described by

(14) $$H\left(I(x,y)\right)=-\sum_{i=0}^{L}p\left(I_{i}\right)\log p\left(I_{i}\right)$$

where *p* is the probability of pixel value $I_{i}$ occurring within I(x,y), and *L* is the maximum pixel value within I(x,y). Shannon entropy provides a global quantitative assessment, considering only global statistical information of I(x,y) while discarding the spatial distribution information. This means that two images with the same Shannon entropy value (quantitative assessment) can be completely different when qualitatively assessed by human vision [28,55]. Thus, Shannon entropy cannot be used to infer image structure. However, when Shannon entropy is used in combination with wavelet decomposition, as shown in Equation (15), Shannon entropy values can be representative of image structure (in particular edge structure within an image). To combine Shannon entropy with wavelet decomposition, I(x,y) is substituted for the combined and normalized wavelet data and the Shannon entropy value is calculated at each decomposition level (*j*). Thus, I(x,y) = ℧(j,m,n) resulting in

(15) $$H\left(℧(j,m,n)\right)=-\sum_{i=0}^{L}p\left({℧}_{i}\right)\log p\left({℧}_{i}\right)$$

**Figure 4:**
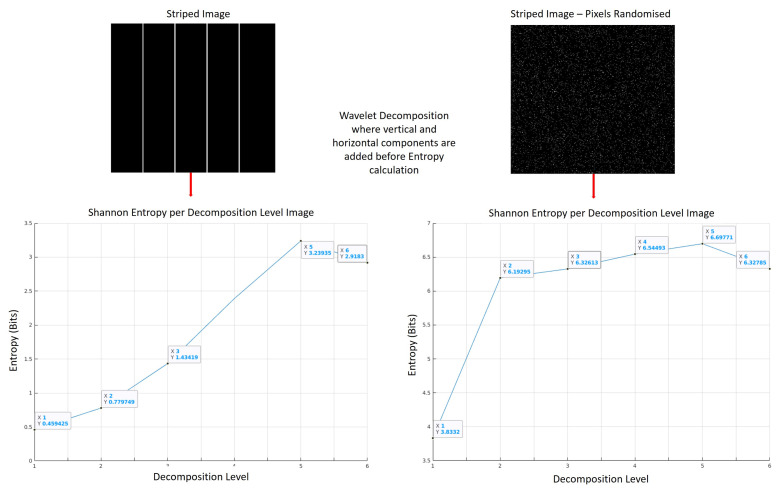
Global structure measurement using Shannon entropy. Both images are 512 × 512 pixels in size.

The decomposition step successfully isolates all gradient magnitudes and directions into individual image frequency and orientation layers. It is assumed that each decomposition level has an assumed level of image structure which can be measured globally using Shannon entropy. Lower values of entropy indicate a high level of redundancy within a given dataset. In the case of our wavelet decomposition levels, a low entropy value will indicate the presence of repetitive sequences and clusters within a decomposition level, indicating that a particular level has a high level of structure. By comparing the Shannon entropy value for each of the decomposition levels, we can form a quantitative assessment of which the decomposition level has the greatest image structure. The $H\left(℧(j,m,n)\right)$ with the lowest Shannon entropy value is expected to have the most structure and therefore the most useful edge data as shown in Figure 4.

Figure 4 contains two images: a striped black and white image and the same image with the pixels randomly shuffled. Figure 4 identifies that the first structured image generates lower entropy values. The randomly shuffled image contains the same statistical distribution as the first image; however, it generates higher entropy indicating less image structure. Without applying wavelet decomposition before the Shannon entropy calculation, both images generate the same Shannon entropy value which provides no quantitative assessment of image structure.

**Figure 5:**
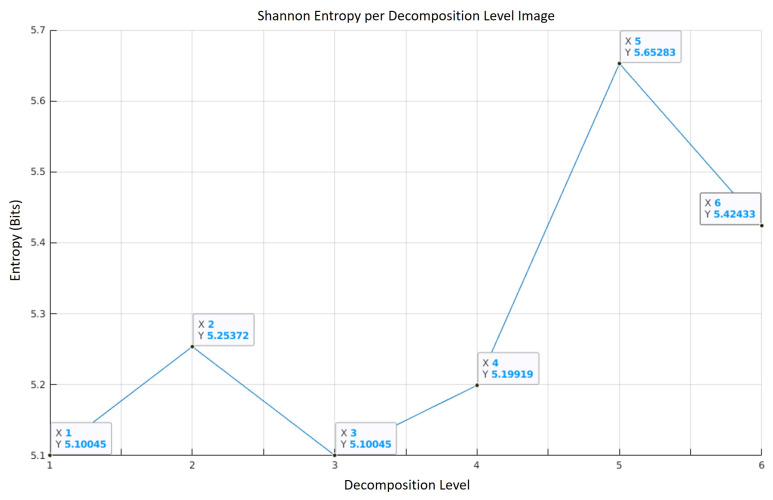
Entropy per decomposition level where vertical and horizontal components are added and normalised using the test image from Figure 3.

Wavelet decomposition level selection discards edge data that contains a low level of structure (high entropy and thus low redundancy). The use of Shannon entropy to select which decomposition levels are kept or discarded means that we are identifying which level contains the highest level of redundancy, and therefore the highest level of edge structure. It is therefore assumed that discarded decomposition levels will only contain minimal edge structure that do not contribute significantly to the definition of edge structure. This helps enable the algorithm to provide an efficient and low redundancy result. Figure 5 provides an example of Shannon entropy values for ℧(j,m,n) when the example image (“House”) from Figure 3 is used.

Note that we denote the optimal wavelet decomposition level as ℧(β,m,n). The optimal wavelet decomposition Level will be the level that has the lowest entropy value. In the case of Figure 3, ℧(β,m,n)=℧(1,m,n).

### 3.3. Entropy Thresholding

Once wavelet decomposition level selection is completed, we are able to identify the optimal edge detection image. Depending on the wavelet chosen for decomposition, artifacts may be generated in the edge detection image. The generation of artifacts depends on the properties of the wavelet chosen and how it convolves with the input image. To overcome these artifacts and enhance the quality of the edge detection image, a variable threshold Λ is applied to the image:

(16) $$\begin{array}{cc} \; & \forall m,n\in ℧(\beta ,m,n)\\ & ℧\left(\beta ,m,n\right)=\left\{\begin{array}{c} ℧(\beta ,m,n)\;\mathrm{if}\;℧(\beta ,m,n)\geq \Lambda \\ 0\;\;\;\;\;\mathrm{if}\;℧(\beta ,m,n)<\Lambda \end{array}\right. \end{array}$$

where Λ is the threshold value and varies in the range of 0≤Λ≤1.

**Figure 6:**
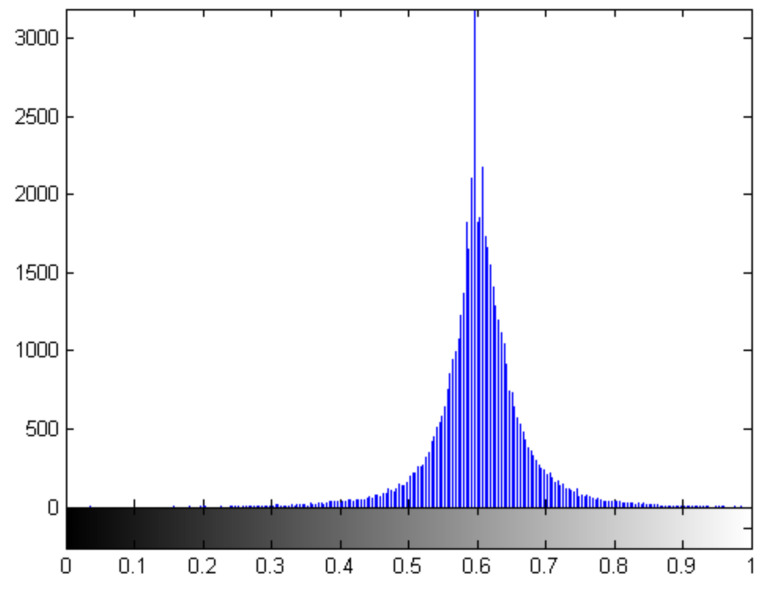
Pixel histogram for ℧(β,m,n) using the test image.

A number of methods [28,33,55] exist for the selection of the most appropriate threshold value within an image to detect edges. Typically, these methods find a threshold based on the histogram of a grey scale image which is multi-modal. Due to the wavelet decomposition used in the selection of ℧(β,m,n), the image we are thresholding is not multi-modal. In our case ℧(β,m,n), selected during wavelet decomposition level selection, will have a Gaussian like histogram distribution as shown in Figure 6.

**Figure 7:**
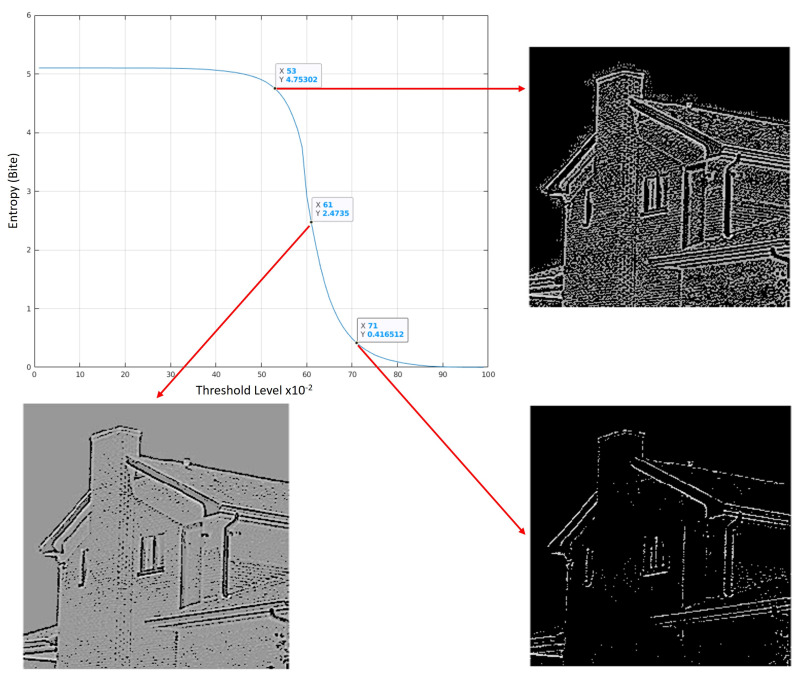
Shannon entropy curve for threshold selection and Coiflet wavelet. Output images are 258 × 258 pixels in size.

From Figure 6, we can see that pixel sequences from I(x,y) that correlate well with $\psi_{\beta ,m,n}^{H}$ and $\psi_{\beta ,m,n}^{V}$ will result in a value closer to one. Likewise, pixel sequences from I(x,y) that don’t correlate well with $\psi_{\beta ,m,n}^{H}$ and $\psi_{\beta ,m,n}^{V}$ will result in a value closer to zero. To determine the optimal threshold, we generate a Shannon entropy curve for ℧(β,m,n) which is done by changing the threshold (Λ) from 0.1 to 1 in 0.1 increments and, for each increment, we use (14) to calculate the Shannon entropy value. Figure 7 shows the Shannon entropy curve for ℧(β,m,n) generated for the test image from the previous step.

**Figure 8:**
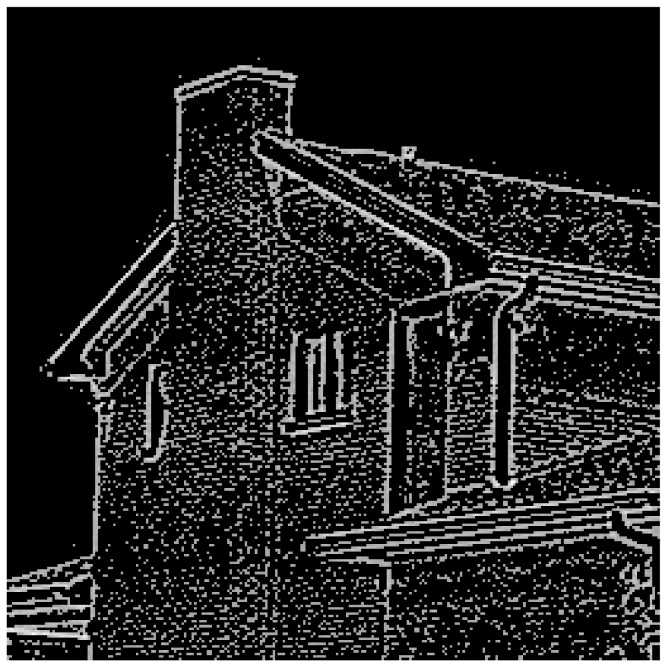
The resultant image (258 × 258 pixels) with Coiflet, j = 1, Λ = 0.635.

Figure 7 shows that the optimal threshold value will exist somewhere between the inflection point (Λ = 0.61) and the minimum entropy value (Λ = 1). The inflection point provides a suitable image but will contain a number of artifacts. The artifacts reduce as the Shannon entropy value tends towards zero, however, so does the edge data. An application of the algorithm must compromise between edge detail and the number of artifacts shown in the output image. Figure 8 shows the final output using the “House” test image with a Λ of 0.635.

## 4. Results

**Figure 9:**
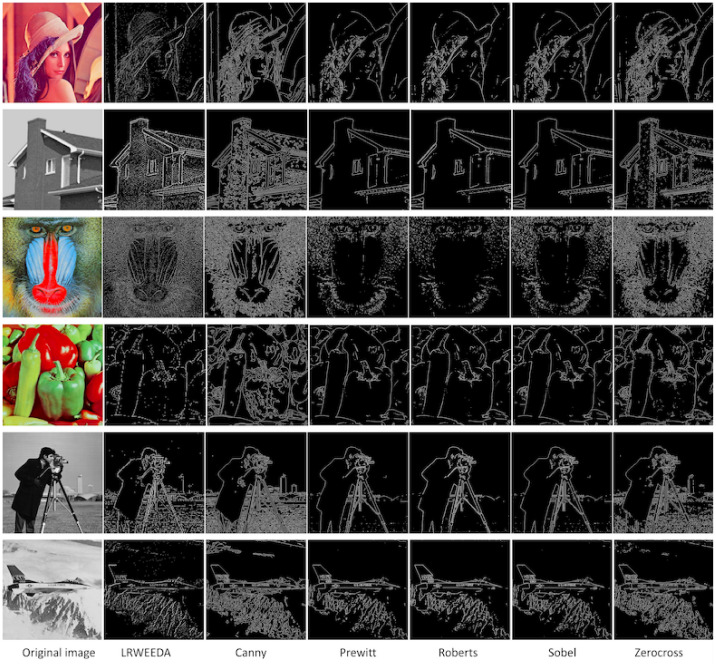
The original image is shown in the first column of the image grid. Other columns correspond to different edge detection algorithms, and the algorithm name is mentioned in the bottom of the column. For the LRWEEDA algorithm, the following parameters were used (from the first row to the last row): (1) “Lena” image: LRWEEDA (output image size of 256 × 256 pixels) using Haar, j = 1, Λ = 0.480; (2) “House” image: LRWEEDA (258 × 258 pixels) using Coiflet, j = 1, Λ = 0.635; (3) “Mandril” image: LRWEEDA (258 × 258 pixels) using Coiflet, j = 1, Λ = 0.520; (4) “Peppers” image: LRWEEDA (131 × 131 pixels) using Coiflet, j = 2, Λ = 0.480; (5) “Cameraman” image: LRWEEDA (131 × 131 pixels) using Coiflet, j = 2, Λ = 0.530; and (6) “Jetplane” image: LRWEEDA (256 × 256 pixels) using Haar, j = 1, Λ = 0.555.

The algorithm was tested on a small dataset of six images (Lena, House, Mandril, Peppers, Cameraman and Jetplane). The same six processing sets were used with the Canny, Prewitt, Roberts, Sobel and Zerocross edge detection algorithms. The results can be seen in Figure 9.

**Figure 10:**
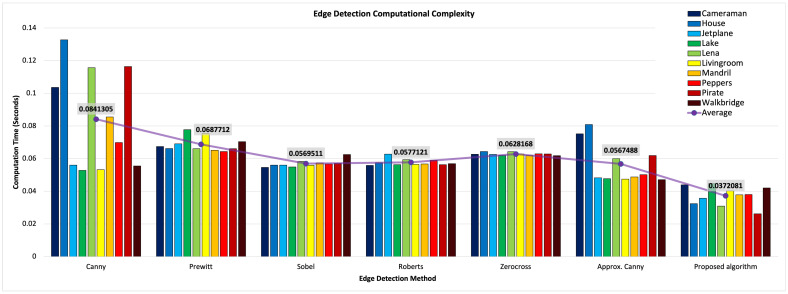
Average computation time for different edge detection algorithms. A dataset of ten images was used for the analysis.

LRWEEDA achieves similar quality edge detection results to the Canny, Prewitt, Roberts, Sobel and Zerocross edge detection algorithms; however, it has achieved this with a significantly reduced pixel count due to the downsampling that occurs in the wavelet decomposition stage. It is important to note that, in each case, the results represent the theoretically optimal image quality that can be achieved with the lowest level of redundant pixels, due to the use of Shannon entropy to detect image structure. In each of the six images, the algorithm calculated the result in approximately 0.1 s and was only bettered in computational performance on the same hardware by the Zerocross method as shown in Figure 10.

Our algorithm provides greater performance in regions where there is a higher level of edge complexity. For instance, it is able to detect edge features within the brick work of the “House” image, the feather in the hat (“Lena” image) and the hairs on the face of the “Mandril” image. The algorithm does not break down in areas of high edge complexity as the algorithm does not include a point clustering step like the Canny edge detection algorithm. This results in LRWEEDA generating false points within the image; however, it reduces the number of false edge artifacts similar to those seen in the brick work of the Canny processed image (see second row, third column of Figure 9). Our algorithm was able to achieve this due to the use of Shannon entropy which enables a simplified threshold technique to be used to reduce false points and therefore noise.

### 4.1. Computational Efficiency

The computational efficiency was tested against five edge detection algorithms using a dataset of ten standard images (all images were unsigned 8 bit grey scale 512 × 512 pixel images). To test computational efficiency, the execution time for each edge detection algorithm was recorded. The proposed algorithm is shown in Algorithm 1.

**Algorithm 1:** LRWEEDA edge detection

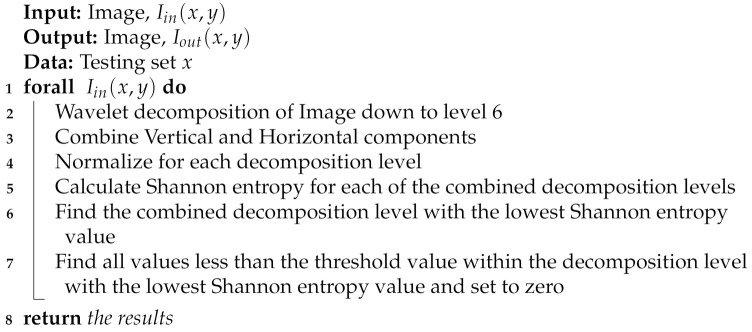



Figure 10 shows that the proposed algorithm executes faster than existing existing edge detection techniques (i.e., Canny, Prewitt, Sobel, Roberts, Zerocross and approximate Canny). The Canny edge detector method takes approximately double the computational time required by the proposed method. The proposed algorithm is designed for scalability and parallel stream processing. Implementation of LRWEEDA on an ASIC, GPU or FPGA medium would result in significantly reduced computation times and low memory usage sufficient for real world applications. The edge detection output for each edge detection method is a 512 × 512 pixel image except for LRWEEDA, which produces a reduced pixel output dependent on the decomposition level selected (17). Edge detector algorithms are typically used as the first step in a larger image processing task. LRWEEDA’s ability to generate an edge detection image with a reduced pixel output means that processing time for further image processing stages can be significantly reduced by applying pixel reduction

(17) $$\kappa =\frac{N}{2^{2j}}$$

where *N* are the number of pixels in the original input image and *j* is the selected decomposition level.

### 4.2. Noise Resilience

**Figure 11:**
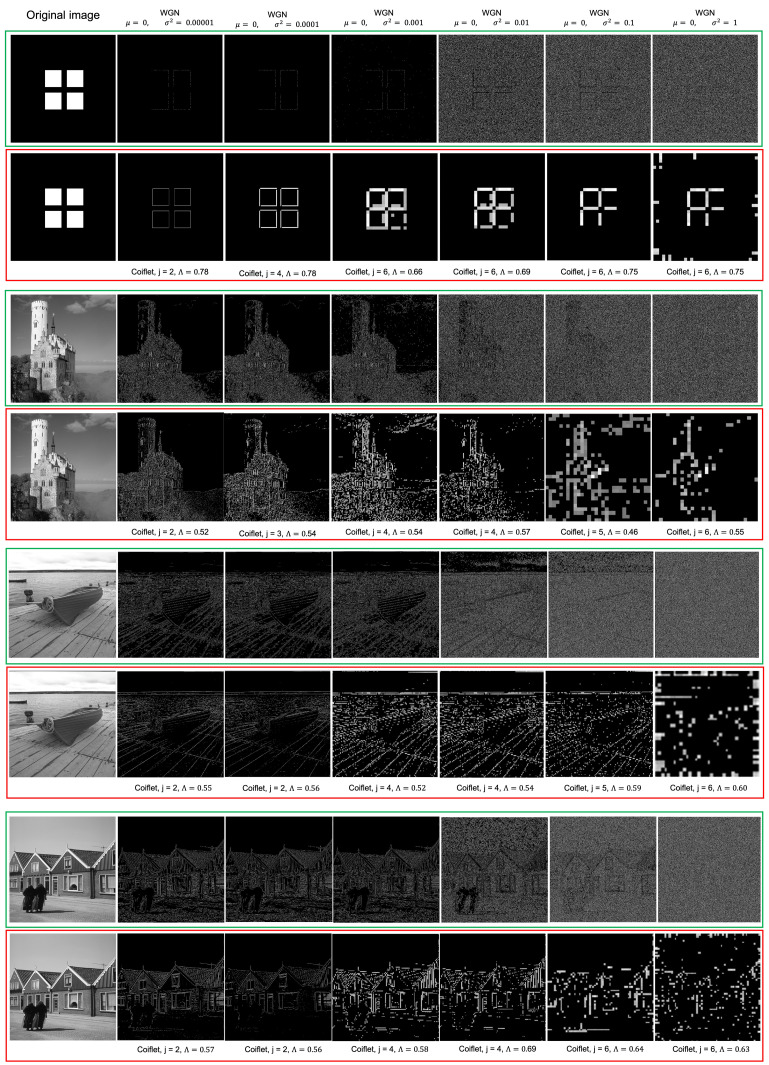
The original image is shown in the first column of the image grid. Other columns correspond to different noise levels. The first, third, fifth, and seventh rows correspond to Canny processed images (grouped in green) and the second, fourth, sixth and eighth rows correspond to LRWEEDA processed images (grouped in red). The corresponding LRWEEDA parameters are shown below the images of red groups. The LRWEEDA processed output images sizes are 483 × 483 pixels when j=2; 244 × 244 pixels when j=3; 124 × 124 pixels when j=4; 64 × 64 pixels when j=5 and 34 × 34 pixels when j=6.

The ability for the proposed method to quantitatively measure structure within the image means that it is inherently more resilient to the effects of noise. To determine the resilience of LRWEEDA to noise, White Gaussian Noise (WGN) with a Probability Density Function (PDF) of

(18) $$\frac{1}{\sigma \sqrt{2\pi }}e^{-\frac{{\left(z-\mu \right)}^{2}}{2\sigma^{2}}}$$

where *z* represents the grey level, μ the mean value and σ the standard deviation was added to two images. WGN with a μ of 0 and variance ($\sigma^{2}$) of 0.00001, 0.0001, 0.001, 0.01, 0.1 and 1 was added to both images before being processed with the Canny and LRWEEDA algorithms. An artificial square image (first row, first column of Figure 11), a real world image (third row, first column of Figure 11), and two images from BSDS500 dataset [9] (fifth and seventh row, first column of Figure 11) were used as the four images. All images were 8 bit gray scale 1920 × 1920 pixel images, and the results can be found in Figure 11.

In all image test cases, as the noise levels increased (increasing σ), LRWEEDA used high decomposition levels (*j*) as ℧(β,m,n). This is expected as increasing noise levels result in an increase of noise away from the higher frequencies and towards the lower image frequencies. The increased noise at the lower image frequencies therefore begins to reduce the amount of structure now appearing at lower decomposition levels (lower *j* values) which raises their entropy value. Therefore, LRWEEDA can be used to find the image frequencies (using the Wavelet scale function) which contains the lowest level of noise corruption whilst containing the highest level of edge structure. This behaviour within the algorithm is closely linked to wavelet properties which have been heavily exploited in image processing for image denoising processes [59].

Using the artificial square image (see first row, first column of Figure 11), LRWEEDA was able to successfully detect edge data for all values of $\sigma^{2}$; however, the quality of detected edge data decreased as $\sigma^{2}$ increased. In contrast, Canny showed degraded detection of edge data from $\sigma^{2}$ = 0.00001 to 0.001 and shows severely degraded edge data for $\sigma^{2}>$ 0.001. Whilst LRWEEDA did have degradation to the resolution of the edge image as $\sigma^{2}$ increased, unlike Canny, it did not have a significant increase in edge artifacts caused by the false detection of edges due to noise. This occurs due to LRWEEDA using higher decomposition levels (*j*) as ℧(β,m,n) which results in a low resolution edge image; however, LRWEEDA is able to determine which ℧(j,m,n) has the lowest level of noise but the highest level of structure (lowest entropy value) and use this as ℧(β,m,n). Canny, on the other hand, relies on its initial blurring function to remove and average out the noise within an image which is not as effective in the removal of WGN. Canny is further affected by the clustering of points to give edge continuity. As a result of the clustering component of the Canny algorithm, edge artifacts increase as false detection of edges caused by the noise are clustered together creating a further reduction in edge detection performance.

Applied to the other images, LRWEEDA was able to detect edge data for all values of $\sigma^{2}$; however, the number and quality of detected edge data significantly decreased as $\sigma^{2}$ increased. In contrast, Canny showed degraded detection of edge data from $\sigma^{2}$ = 0.00001 to 0.001 and shows severely degrade edge data for $\sigma^{2}>$ 0.001. LRWEEDA was once again able to determine which ℧(j,m,n) had the lowest level of noise but the highest level of structure (lowest entropy value) and used this as ℧(β,m,n). Canny, on the other hand, relies on its initial blurring function to remove and average out the noise within an image which is not as effective in the removal of WGN; furthermore, Canny was also hindered by its edge clustering properties. It is interesting to note that, in the case of the least amount of noise ($\sigma^{2}$ = 0.00001) (third row, second column and fourth row, second column of Figure 11), LRWEEDA demonstrates a clear improvement in edge detection quality compared to Canny. In particular, LRWEEDA clearly identifies the building and tower structural features with minimal artifacts compared to the Canny algorithm.

### 4.3. Performance against Standard Edge Detection Metrics

**Figure 12:**
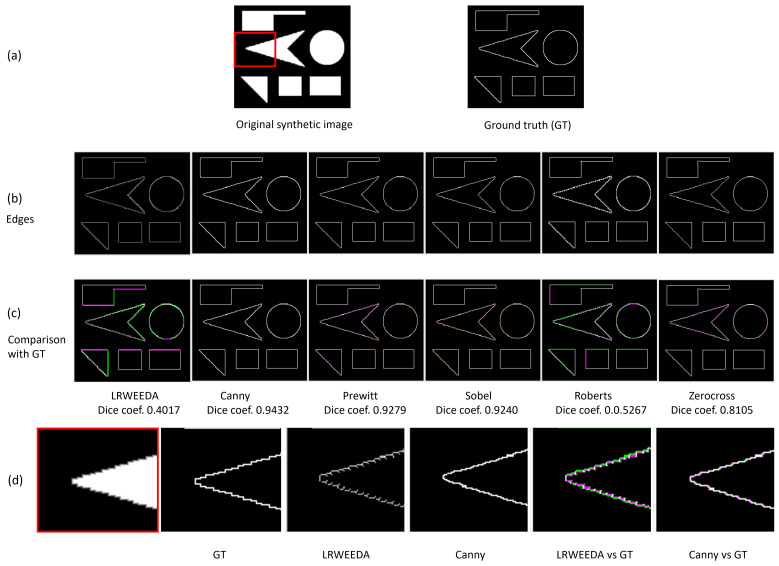
A synthetic image was used to calculate the Dice coefficient of the proposed algorithm. The original synthetic image (270 × 238 pixels) and the ground truth edges (270 × 238 pixels) are shown in (**a**). The edges obtained by LRWEEDA, Canny, Prewitt, Sobel, Roberts and Zero cross algorithms are shown from left to right in (**b**). In (**b**), all the images are 270 × 238 pixels. The edges were compared with the ground truth in (**c**). The edges calculated by each algorithm and the ground truth edges are shown in green and purple, respectively. The overlap between the calculated edges and the ground truth is shown in white. The Dice coefficient for each algorithm is shown under each image. The red color box in (**a**) is enlarged for the analysis purpose in (**d**).

We calculated the Dice coefficient of the proposed algorithm using a synthetic image (see Figure 12).

• The Dice similarity coefficient (DSC), also called the $F_{1}$ score, is used to evaluate the similarity of two samples. It is calculated as the harmonic mean of the precision and the recall as

(19) $$DSC=\frac{TP}{TP+\frac{1}{2}\left(FP+FN\right)}$$

or

(20) $$F_{1}=2\cdot \frac{precision\cdot recall}{precision+recall}$$

where *TP* is the true positive, *FP* is the false positive and *FN* is the false negative.

Dice coefficient returns a value between 0 and 1, where 1 is the highest similarity. To evaluate the edge detection performance, a higher Dice coefficient means more accuracy for the prediction of the edges compared to the ground truth images. The Dice coefficients were calculated for the resultant edge image of each algorithm.

As shown in Figure 12c, LRWEEDA shows the lowest Dice coefficient among the tested algorithms. However, it detects all the edges with additional local image details. In Figure 12d, an enlarged section of the original synthetic image is analyzed. As can be seen from the red bordered image, the original image has saw tooth edges. The proposed algorithm is sensitive to minor local image details and hence shows more local details in the resultant image (LRWEEDA’s ability to detect complex local edges (Figure 9) was discussed in the beginning of this section). When compared to the ground truth, these local edges are considered as noise as they were not represented in the ground truth. Therefore, in this analysis, LRWEEDA shows a lower Dice coefficient.

We further analyzed the performance of the proposed algorithm against four standard edge detection metrics. In addition to Dice coefficient, we tested our algorithm with a boundary F1 score (BFS) [60], Jaccard coefficient (JC) [60], and Pratt’s figure of merit (FOM) [61] metrics to analyze the performance. The metrics are summarized below.

- The Boundary F1 score is defined as the harmonic mean (F1-measure) of the precision and recall values which measure the matching weight for the predicted boundary and the ground truth boundary, as
  (21) BFS=2·precision·recall/(recall+precision).
- The Jaccard coefficient for two sets is defined as the size of the intersection of the two sets divided by the size of their union as
  (22) $$JC=\frac{TP}{\left(TP+FP+FN\right)}.$$
- Pratt’s FOM uses Euclidean distance to compare two edge images [60]. It multiplies a scale factor ∝ to the Euclidean distance calculated between the two images to penalize displaced edges, as
  (23) $$Pratt^{\prime }sFOM=\frac{1}{max(I_{A},I_{B})}\sum_{i=1}^{I_{A}}\frac{1}{1+\propto d_{i}^{2}}$$
  where $I_{A}$, $I_{I}$, and *d* are, respectively, the detected edges, the ideal edges, the distance between the actual and the ideal edges.

**Figure 13:**
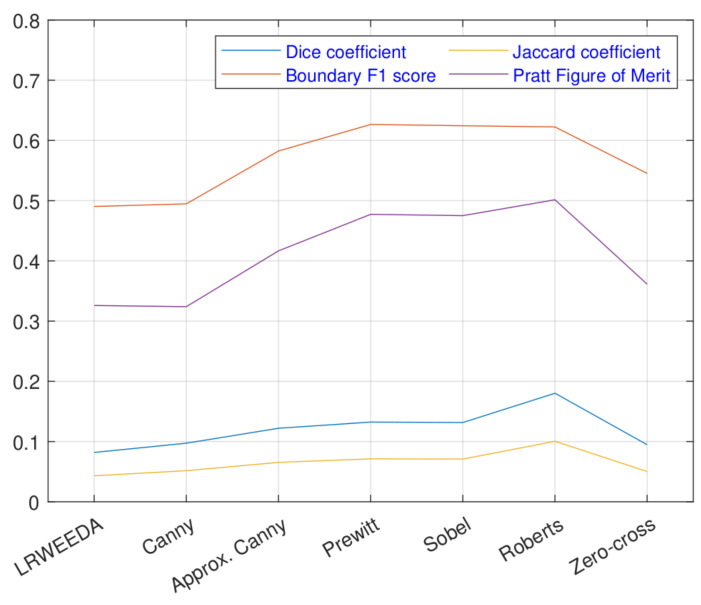
Comparison of the performance metrics.

A test dataset of 20 images were selected from the BSDS500 dataset. The performance metrics of the dataset are compared in Figure 13. As shown in Figure 13, LRWEEDA and Canny metrics are closer to each other. Overall, LRWEEDA, Canny and Zero-cross algorithms show roughly similar performance. LRWEEDA does not show superior performance over the other algorithms in terms of these performance metrics. However, its performance is within the range of the Canny and Zero-cross while representing complex local edges.

We ran all the experiments in MATLAB using a 2.60 GHz laptop computer. The MATLAB functions of ‘canny’, ‘approxcanny’, ‘prewitt’, ‘sobel’, ‘roberts’, and ‘zerocross’ were used for the comparison. Four experiments were conducted to validate the findings with the following image sets:

- Qualitative results of the proposed algorithm were obtained and compared with similar edge detection algorithms (Figure 9)
- Ten images were used to calculate the average processing times of the algorithms (Figure 10).
- Noise resilience of the proposed algorithm was analyzed by using four images and compared with Canny (Figure 9).
- Performance against standard edge detection metrics were calculated using a synthetic image and 20 images from BSDS500 dataset (Figure 12 and Figure 13).

Overall, the performance analysis in Figure 10 and Figure 13 show that LRWEEDA’s edge detection performance is within the performance range of popular edge detection algorithms while its processing time is lower compared to other similar algorithms.

## 5. Conclusions

We have developed a method for edge detection using a combination of the Wavelet transform, Shannon entropy and thresholding. The assumption of structure within a wavelet decomposition enabled the use of Shannon entropy as a measure of global image structure. Our work has resulted in a new edge detection method, known as the Low Redundancy Wavelet Entropy Edge Detection Algorithm that has been developed as an efficient, low redundancy, robust edge detection algorithm well suited to real-time image processing applications. Our analysis shows that LRWEEDA can generate similar or better edge detection results with significantly less output data being generated. Experiments were conducted on six standard image processing sets and we compared the performance or LRWEEDA to five popular edge detection algorithms. High resolution images with artificial and real world scenes were used to assess the noise resilience of the proposed algorithm.

For future work, we are focusing on further reducing the processing time of the algorithm and improving the threshold selection on the Shannon entropy curve. The low processing time of the proposed algorithm should be more appealing for high speed edge detection applications.

## Data Availability

Not applicable.
